# Rapid Isolation of intact *Salmonella*-containing vacuoles using paramagnetic nanoparticles

**DOI:** 10.1186/s13099-018-0256-7

**Published:** 2018-07-31

**Authors:** Vikash Singh, Peter Schwerk, Karsten Tedin

**Affiliations:** 1 Centre for Infection Medicine, Institute of Microbiology and Epizootics, Free University of Berlin, Robert-von-Ostertag-Str. 7-13, 14163 Berlin, Germany; 2 Department of Pathology, University of Cambridge, Tennis Court Road, Cambridge, CB2 1QP UK

**Keywords:** Nanoparticles, Bacteria containing phagosome, Isolation, Salmonella, Infection, Macrophages

## Abstract

**Background:**

Both typhoidal and non-typhoidal *Salmonella* infections remain a considerable cause of morbidity and mortality globally, and impose a major socio-economic burden worldwide. A key property of all pathogenic *Salmonella* strains is the ability to invade host cells and reside within an intracellular, vacuolar compartment called the *Salmonella*-containing vacuole (SCV). Although the SCV is involved in both immune-evasion and intracellular replication and spread within the host, information about the host:pathogen interactions at this interface are limited, in part due to the technical difficulties involved in purification of these vacuoles. While a number of column- or gradient-based methods have been applied, cross-contamination with other host cell organelles or rupture of the labile SCV membrane has further complicated efforts to successfully isolate SCVs.

**Results:**

Here, we report the isolation of intact SCVs using carbon-coated, paramagnetic nanoparticles. The approach permits rapid isolation of intact SCVs from human macrophages in vitro without involving numerous purification steps. Bacteria are pre-labeled with modified nanoparticles prior to infection, and at various times post-infection, host cells are lysed and intact pathogen-containing phagosomes are recovered after application of a mild magnetic field. Purified, intact SCVs isolated using this method were shown to display high levels of co-association of internalized *Salmonella* with the standard SCV markers Rab5 and LAMP-1 using both microscopic and protein based methods.

**Conclusion:**

The method described is highly efficient, robust and permits rapid isolation of intact SCVs from human macrophages without involving numerous purification steps. The method can also be applied to other intracellular pathogens that reside within a vacuole-like compartment within host cells. Future work using the approach should aid in identification and characterization of host factors associated with the membranes of such intracellular pathogens, which could potentially serve as pharmaceutical targets against intracellular pathogens residing within vacuoles.

**Electronic supplementary material:**

The online version of this article (10.1186/s13099-018-0256-7) contains supplementary material, which is available to authorized users.

## Background

*Salmonella enterica* serovars are Gram-negative, facultative intracellular bacteria that infect and colonize vertebrate hosts with outcomes ranging from sub-clinical infections to life-threatening systemic diseases [1]. *Salmonella* infections constitute a global public health burden, not only due to infections by *S*. Typhi, the human-restricted serovar causing human Typhoid, but also due to non-Typhoidal salmonellosis. The non-typhoidal *Salmonella* serovars can differ both in their host specificity and in the pathogenesis in different species [2]. The broad host-range serovar *Salmonella enterica* serovar Typhimurium infects a wide range of hosts, both human and animal, as well as different host cell types, including macrophages and non-phagocytic cells such as the intestinal epithelia [3, 4].

The major *Salmonella* virulence genes involved in host cell invasion and establishment of the intracellular replicative compartment known as the *Salmonella*-containing vacuole (SCV) are encoded within two pathogenicity islands, denoted SPI-1 and SPI-2, respectively [5, 6]. *Salmonella* translocates more than 30 SPI-2 encoded effectors or virulence factors across the SCV membrane, which enable the pathogen to replicate, colonize and establish infection within its host [7]. The maturation of the SCV is characterised by a time-dependent acquisition and loss of various endosomal and lysosomal markers that distinguish it from the lysosomal degradation pathway [8, 9].

A major difficulty in identifying and understanding the various host proteins involved in maturation of pathogen-specific, phagosome-like compartments has been the isolation of intact pathogen-containing phagosomes from host cells. Bouyant density gradient centrifugation has been previously applied, in which latex beads are pre- or post-loaded with phagosomes containing pathogens and purified from fractions taken from the gradients where the latex particles show a discrete banding pattern [10, 11]. However, this method is dependent on separation from within a gradient of vesicles with similar densities, where cross-contamination is often unavoidable. Magnetic cell separation (MACS) and other column-based methods require removal of non-bound material with extensive washing steps, and labile compartments such as phagosomes may lose considerable amounts of material and loosely bound proteins [12, 13]. Uncertainties due to cross-contamination with other cellular compartments or numerous purification steps accompanied by loss of the labile vacuolar membrane has therefore hampered a better characterisation of these intracellular compartments. An improvement has been the application of flow-assisted cell sorting (FACS), in which GFP- or other fluorescently-labelled bacteria are used to infect cells, and after lysis of the cells, the cellular contents are passed through a flow cytometer and fluorescently-labelled components are diverted from the flow-through for collection [14]. However, the shear forces generated often lead to disruption of the phagosomes and the volumes required are often accompanied by inefficient concentration of the samples.

Here, we describe a simple method for successfully isolating intact SCVs by pre-labelling the bacteria with carboxyl-coated paramagnetic nanoparticles prior to infection. The method described is rapid, robust and efficient enough to collect intact SCVs for further, downstream analyses such as Western blotting, immunofluorescence, and intravacuolar bacteria recovery.

## Results

### Overview of the isolation method

The methodology described for the isolation of intact *Salmonella*-containing vacuoles (SCVs) makes use of paramagnetic nanoparticles that are between 10 and 50 nm in diameter. The nanoparticles consist of a central metal cobalt ion, which is carbon-coated to avoid possible metal toxicity, and further modified with carboxyl functional groups to provide a charge for non-covalent binding to the bacterial (target) surface.

**Fig. 1:**
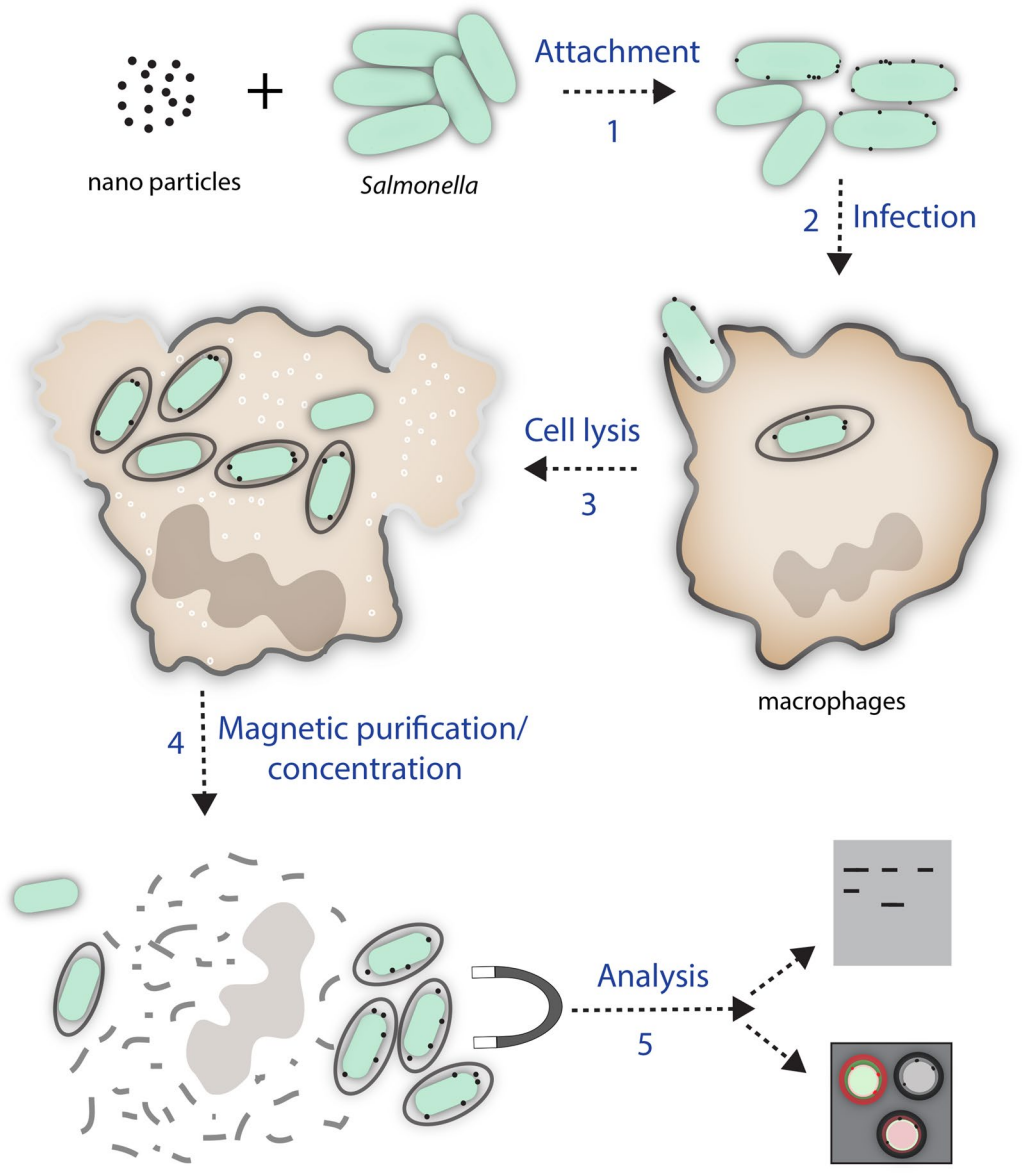
Overview of the method. Carboxyl-coated paramagnetic nanoparticles are incubated with bacteria for 30 min with shaking (1). The paramagnetic nanoparticle-labelled *Salmonella* were then used to infect the human macrophage like cell line THP-1. The internalization of the bacteria is synchronized by a short centrifugation step (2). Internalized *Salmonella* establish an intracellular SCV (3). At different time points, macrophage are lysed using a mild detergent containing 2% sucrose solution (4), and the bacteria are recovered by application of a magnetic field (5). The recovered bacteria are then analysed by standard methods such as immunofluorescense and Western blotting for the presence or absence of standard SCV markers

Unbound nanoparticles are removed by passing the mixture through a 0.2 μm filter, which allows free passage of non-bound nanoparticles. After washing of the retained bacteria, the filter is inverted and the nanoparticle-tagged bacteria are recovered by a back wash with buffer. The recovered bacteria are then used to infect host cells using standard gentamicin-protection (invasion) assays [15]. At different time points post-infection, the infected host cells are lysed and bacteria are recovered in the presence of a magnetic field. An overview of the method is shown in Fig. 1. The recovered bacterial samples are then stained with antibodies specific to host cell proteins known to co-localise with the SCV membrane such as Rab5, EEA1 or LAMP-1 to verify the presence of intact vacuolar membranes prior to further characterisation.

### Non-covalent attachment of carboxyl-coated magnetic nanoparticles to the bacterial surface

**Fig. 2:**
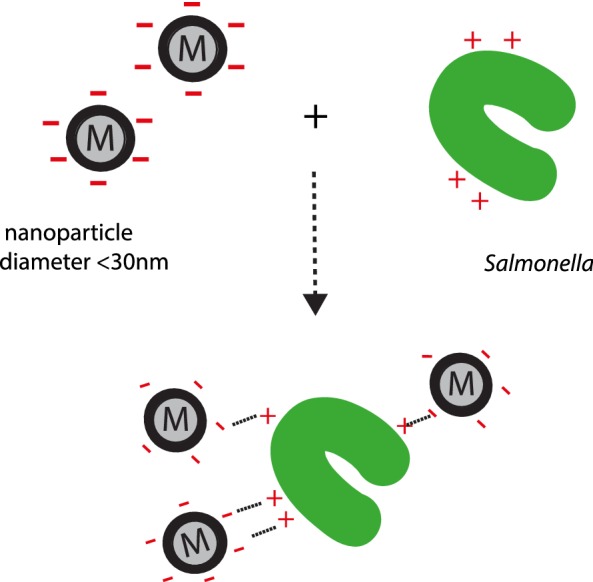
Attachment of carboxyl coated magnetic nanoparticles at the bacterial surface. Schematic representation of the chemistry involved behind the attachment of carboxyl containing nanoparticles on to the bacterial surface via electrostatic bonding

Bacterial attachment to any surface is related to surface charges both on the bacteria as well as on the substratum or target of attachment. Dickson et al. [16] characterized surface charges and their correlation with bacterial attachment. In that study, it was found that for *Salmonella* Typhimurium, the bacterial surface showed an overall net negative charge with r/e (−) 9.47 to r/e (+) 4.78, consistent with earlier studies [17]. Therefore, although the bacterial cell surface has generally an overall negative net charge, there are regions of positive charge present as well. We considered that an excessive labeling of the exterior surface of the bacteria with positively charged particles might result in detrimental effects on either host cell invasion or cytotoxic effects on the host cells. Although the nanoparticles used are much smaller than the average bacterial cell (1–2 μm), excessive particle binding to the bacteria could affect the function of the type III secretion systems, or *Salmonella’s* natural trafficking within the cell. We therefore chose to take advantage of the small, but significant, positive charges on to the bacterial surface to label the bacteria. For this purpose, we used paramagnetic, carbon-coated cobalt nanoparticles of diameter 10–50 nm, with a carboxyl functional group attached to their surface, thus providing a net negative charge for attachment via electrostatic interactions [18] to positively charged regions on the cell surface of *Salmonella*. A schematic representation for the attachment is shown in Fig. 2.

Carboxyl-coated nanoparticles were first sonicated to disperse aggregates and incubated with bacteria suspended in 1× PBS, at a ratio of approximately 5:1 of nanoparticles:bacteria at 37 °C for 20 min with shaking. The bacterial suspensions were then filtered through a 0.2 μm filter fitted to a 5 ml syringe. The filters were washed twice with two volumes of PBS to remove unbound nanoparticles. The filters retaining the bacteria were then inverted and the bacteria were recovered in 1 ml of PBS collected into a sterile microfuge tube. The recovered bacteria were then used for either infection of cell cultures of THP-1 human monocyte/macrophage-like cells (ATCC TIB-202), or processed for transmission electron microscopy.

### Paramagnetic labeling of *Salmonella* does not impair bacterial viability or invasion capabilities

**Fig. 3:**
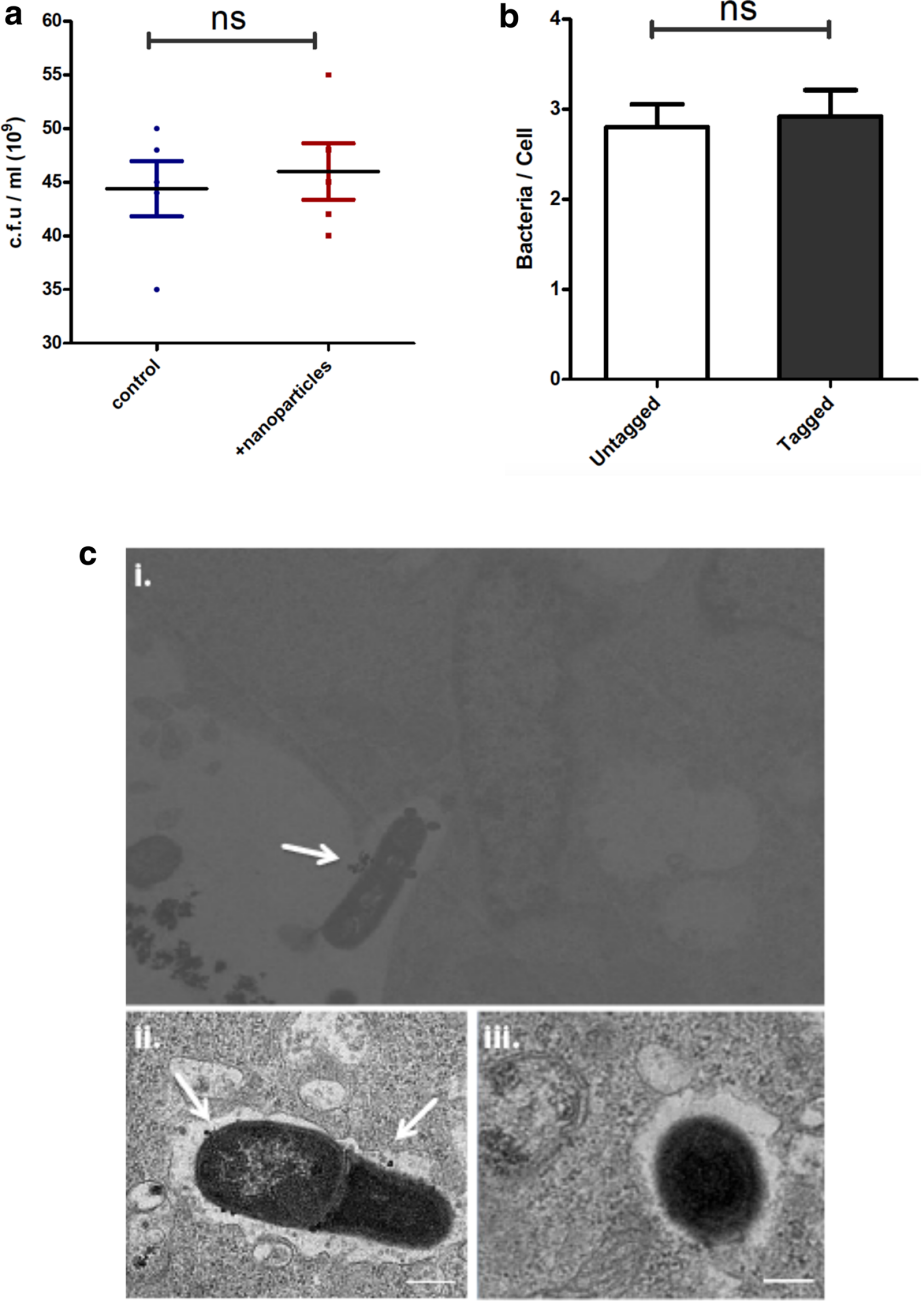
Nanoparticle attachment does not impair bacterial viability or invasion capability. The attachment of nanoparticles on bacterial surface had no significant effect on the viability of *Salmonella* as determined by bacterial c.f.u determinations before and after incubation with nanoparticles (**a**). The nanoparticles did not compromise the ability of *Salmonella* to invade non-phagocytic, intestinal epithelial cells (**b**). Representative TEM images showing nanoparticle-bound *Salmonella* invading THP-1 cells **c** (**i**). Post-internalization, the bacteria retain the nanoparticles within the SCV, indicated by arrows **c** (**ii**), in contrast to non-labelled bacteria **c** (**iii**)

To verify that the attachment or interaction of *Salmonella* with the nanoparticles had no significant effect on the viability of the bacteria, we determined the colony forming units (c.f.u) of *Salmonella* before and after incubation with magnetic nanoparticles at 37 °C. Sonicated nanoparticles were incubated with bacterial suspensions at a ratios of 5:1 or 10:1(nanoparticles:bacteria) and incubated at 37 °C for 40 min with shaking. Samples were taken for c.f.u determinations before and after incubation in order to determine possible effects of the nanoparticles on the viability of *Salmonella*. As shown in Fig. 3a, incubation with nanoparticles had no significant effects on their c.f.u indicating that the attachment of these paramagnetic nanoparticles was not detrimental to the bacteria.

We then determined whether the attachment of nanoparticles to the *Salmonella* surface would affect the invasion process of *Salmonella*. For this purpose, we infected non-phagocytic, intestinal epithelial cells, with either non-tagged *Salmonella* or nanoparticle-tagged bacteria at an MOI of 5:1 (bacteria:epithelial cells). Invasion ratios were determined by calculating the total intracellular bacteria 2 h post infection (p.i) for the control group and the test group. As shown in Fig. 3b, no significant differences were observed on the invasion rates of *Salmonella*, indicating that the attachment of the nanoparticles did not physically hinder the invasion/uptake process of *Salmonella* in into intestinal epithelial cells. Furthermore, following invasion, intracellular *Salmonella* retained the attached paramagnetic nanoparticles within the *Salmonella* containing vacuole. As shown in Fig. 3c (i), the magnetic nanoparticles showed close association with the bacterial outer surface. As expected for electrostatic interactions with only limited positively charged regions of the bacterial surface, the nanoparticles were not uniformly distributed over the entire bacterial surface, but rather found at only a few localized sites.

### SCV purification and analysis

Following internalization into the host cell, *Salmonella* resides within a membrane-bound compartment referred to as the *Salmonella*-containing vacuole (SCV), the maturation of which requires bacterial effector proteins [19]. The maturation of the SCV into a replicative niche can be followed by the acquisition and/or exclusion of various host cell proteins [20]. At early stages post-invasion, endosomal markers such as Rab5 and early endosome antigen 1 (EEA1) are found at the SCV membrane [21]. As the SCV continues to mature, it acquires late endosomal and lysosomal markers such as LAMP1, some of which are involved in acquisition of nutrients from the host cell cytosol [22].

**Fig. 4:**
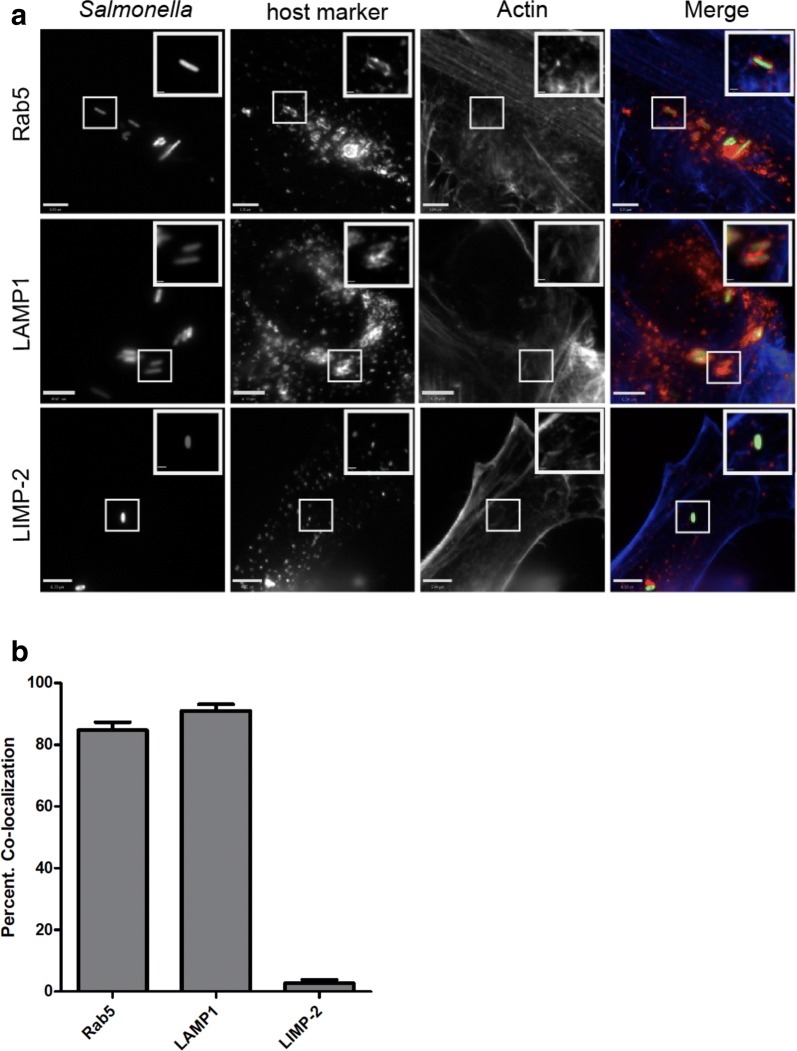
Co-localization studies. Wild-type *Salmonella* expressing GFP (green) were used to infect THP-1 macrophage cells grown on coverslips at a multiplicity of infection (MOI) of 5. The monolayers were fixed 10 min post infection, and Rab5 association determined at and after 6 h and to detect bacterial association with LAMP-1 and LIMP-II. **a** Green, GFP-expressing bacteria co-localized with both Rab5 (red) and LAMP-1 (red), but not with LIMP-II (red), indicating that *Salmonella* is present within an SCV and not lysosomes. **b** Co-localization of intracellular *Salmonella* with both Rab5 and LAMP-1 were also quantified using the Velocity quantification software. The images shown are a representative of at least 25 individual cells per condition performed in duplicates on at least three independent occasions

In order to determine whether pre-labeling of *Salmonella* with paramagnetic nanoparticles might affect association of host cell proteins with the SCV, we infected the THP-1 human macrophage-like cell line with labeled bacteria, and at different time points post-infection, cells were fixed and immuno-stained with anti-Rab5 and anti-LAMP-1 antibodies, both standard markers for the SCV membrane [21]. As depicted in Fig. 4a, nanoparticle-labeled *Salmonella* showed co-localization with both Rab5 and LAMP-1, indicating that the bacteria reside within a phagosome-like vacuole indistinguishable from the SCV, at least with regard to these standard markers [21]. Both Rab5 and LAMP-1 are also well-characterized markers for early endosomes and lysosomes. In order to rule out the possibility that nanoparticle-bound, intracellular *Salmonella* might be affected in SCV maturation and fail to avoid fusion with lysosomes, we chose to use an additional marker specific for lysosomes but not for the SCV [22]. Lysosomal integral membrane protein-2 (LIMP-2), is a member of CD36 superfamily of scavenger receptors [23] and plays an important role in lysosomal membrane organization. LIMP-2 serves as a receptor for lysosomal mannose-6-phosphate-independent targeting of β-glucocerebrosidase [24]. As shown in Fig. 4a, intracellular *Salmonella* does not co-localize with anti-LIMP-2 antibodies thus ruling out lysosomal interaction with the SCV.

**Fig. 5:**
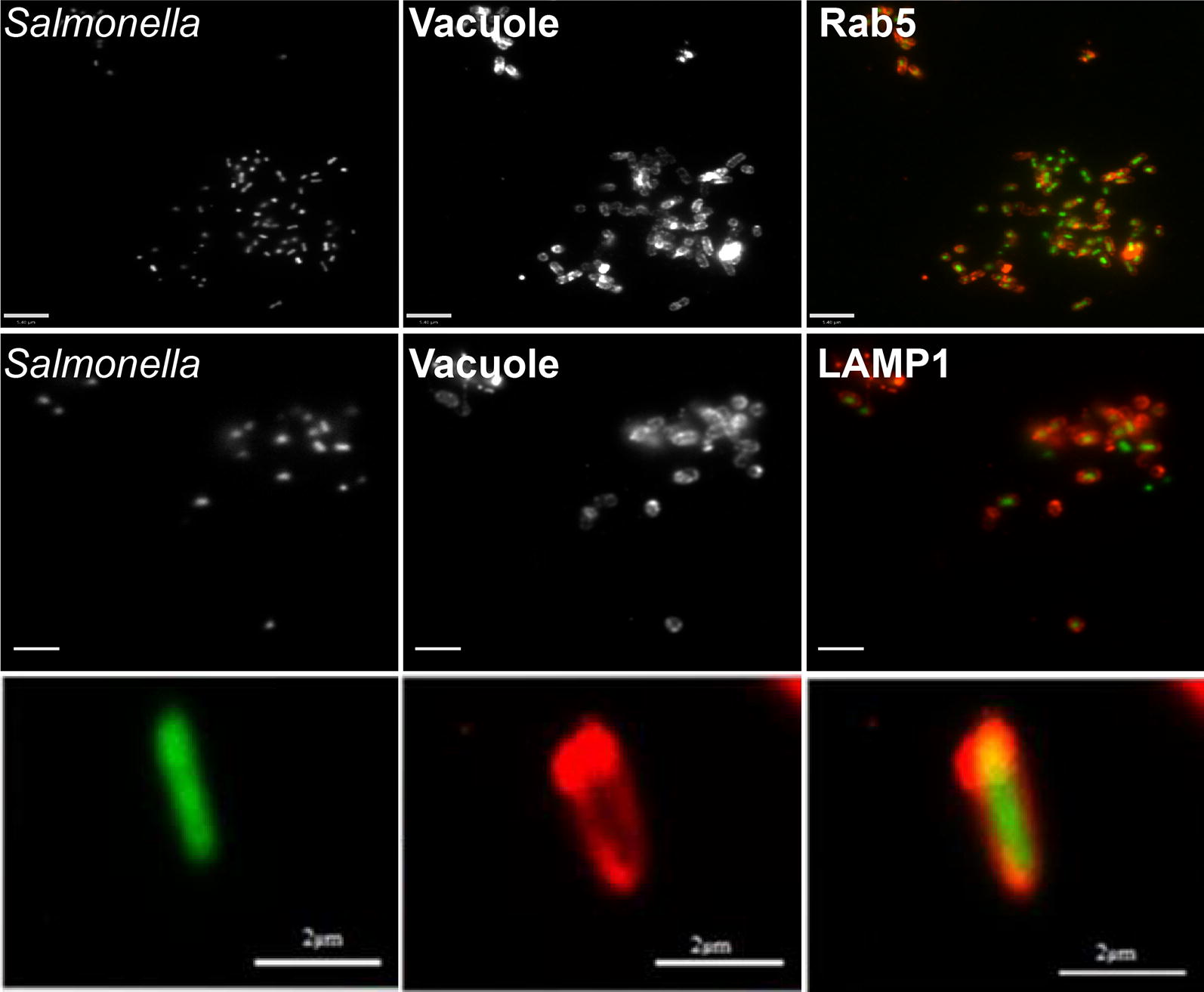
Microscopic analysis of purified SCVs. Bacteria recovered by application of a magnetic field after lysis of THP-1 macrophages were incubated in presence of antibodies against the standard SCV markers Rab5 and LAMP-1. GFP-expressing *Salmonella* show exterior labelling with both Rab5 and LAMP-1 indicating intact, isolated SCVs. The bottom panel shows a close-up of an individual SCV showing a single bacterium (green), LAMP-1 staining (red)

Finally, to determine whether pre-labeling of *Salmonella* with paramagnetic nanoparticles might be used to purify intracellular *Salmonella*-containing vacuoles, host cells were infected, and later lysed for recovery of intracellular *Salmonella* in the presence of a magnetic field. Cell lysates were then placed in a magnetic field and non-bound material was carefully removed, discarded and replaced by 1 ml of a 1× PBS/2% sucrose solution. The recovered material was then further processed by incubating in the presence of anti-Rab5 and anti-LAMP-1 antibodies for microscopy or subjecting aliquots to SDS-PAGE followed by Western blotting analysis. As shown in Fig. 5, GFP-expressing *Salmonella* recovered in this manner from cell lysates showed exterior labeling with both Rab5 and LAMP-1, consistent with the presence of intact SCV membranes as previously shown in Fig. 4a, where 90–95% of intracellular *Salmonella* showed co-localization with both these well characterized SCV markers (Fig. 4b). Control experiments using the secondary antibodies alone showed no co-localization with purified SCVs or labeling of the GFP-expressing *Salmonella* (Additional file 1: Fig. S1).

### Western blot analysis

**Fig. 6:**
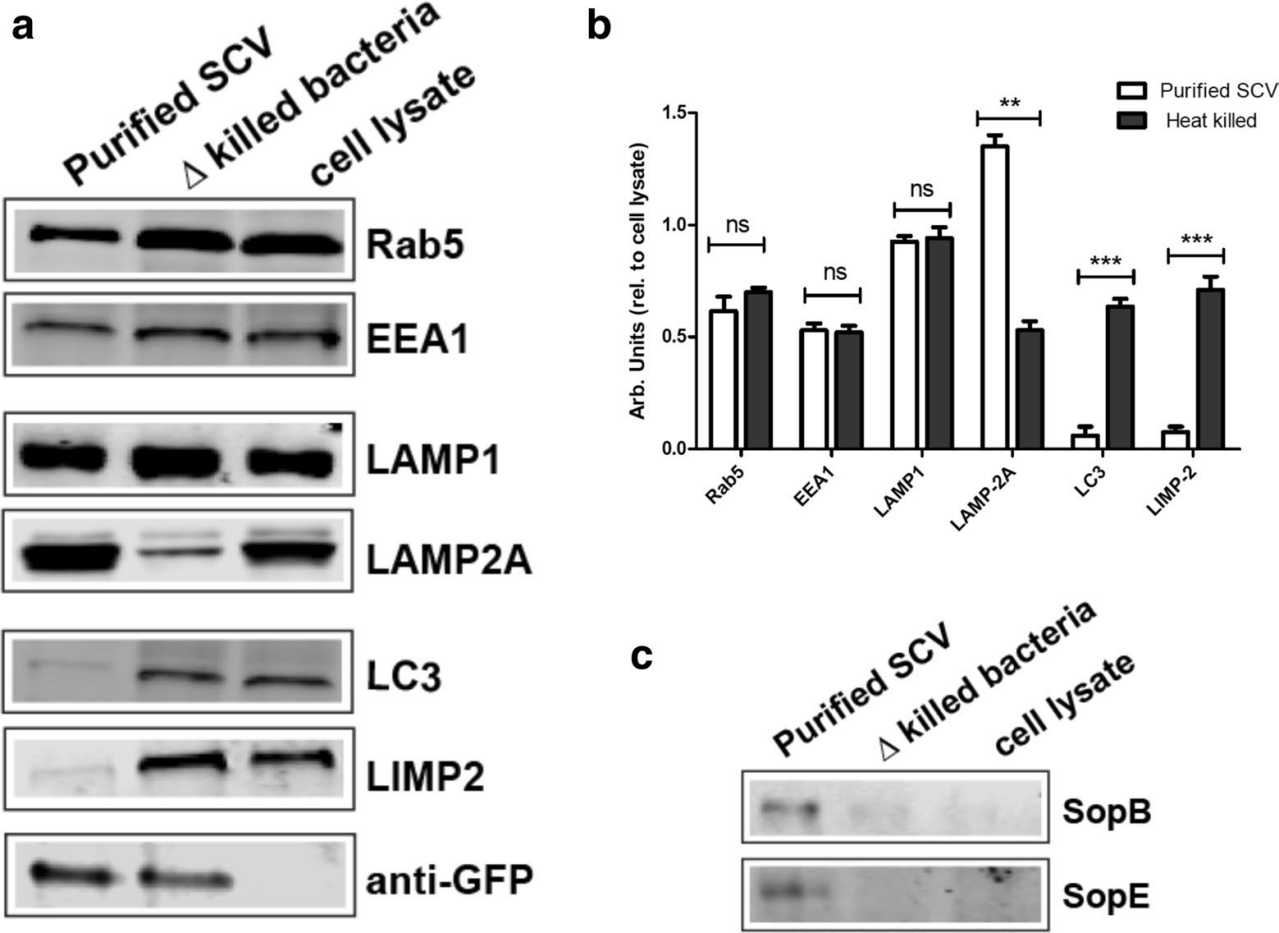
Western blot analysis of purified SCV. Purified SCVs recovered from THP-1 macrophage infected with either live (SCV) or heat-killed (killed) GFP-expressing *Salmonella* were subjected to SDS-PAGE and Western blotting using antibodies against the indicated host proteins. Purified SCVs show the presence of both early (Rab5 and EEA1) and late (LAMP-1 and LAMP-2A) SCV markers but not LIMP-II which is present in heat (killed) group **a** indicating the purified SCVs are devoid of lysosomal contamination. Loading controls consisted of a constant amount of uninfected host cell lysates used as an internal standard for the quantification. **b** Quantification of the relative amounts of the indicated host proteins present in SCVs purified from macrophage infected with either live or heat-killed *Salmonella*. Bacterial effectors SopE and SopB were found exclusively only on purified SCVs (**c**). Signal intensities were determined relative to a constant amount of non-infected cell lysates. The data shown are the means and standard error of at least two, independent experiments. Statistical significance was determined by a two-way Annova, where *p *> 0.5 is considered non-significant (ns); **p < 0.01; ***p < 0.001

To verify the results as observed by microscopic analysis of the purified SCVs, we also performed Western blotting for SCV-associated host proteins using the purified SCV preparations. As a control, nanoparticle-bound *Salmonella* were heat-killed, and incubated with macrophage for uptake by phagocytosis. The SCV preparations were then subjected to SDS-PAGE followed by Western blotting against various lysosomal markers. As expected, SCV markers such as Rab5 and EEA1 (early SCV markers) and LAMP-1 were shown to be present on purified SCV preparations. As previously reported, LAMP-2A was found to be enriched on purified SCVs compared to heat-killed *Salmonella* [22]. In contrast, association with LIMP-2 and LC3 was significantly reduced or absent compared to control phagosomes containing dead *Salmonella* (Fig. 6a). Control preparations using heat-killed *Salmonella* showed the presence of all lysosomal markers, as expected for mature lysosomes. The presence or absence of these markers both on purified SCVs as well as heat-killed *Salmonella* were also quantified as shown in Fig. 6b. The values in arbitrary units represent band density relative to that of cell lysates.

Finally, we also verified the presence of the *Salmonella* effector proteins SopE and SopB, both of which have previously been shown to localize to the SCV membrane, and which play an important role in maintaining and modulating the SCV [25–27]. As can be seen in Fig. 6c, both secreted effector proteins are also found in the membrane of the SCV. These results indicate that the SCVs purified in this manner represent viable, *Salmonella*-derived compartments, as the heat-killed *Salmonella* did not show the presence of these effectors when recovered from macrophage (Fig. 6c).

These results indicated that the purified SCVs showed not only the standard host cell markers associated with the SCV membrane, Rab5 and LAMP-1, but were also essentially devoid of cross-contamination from other endosomal compartments such as lysosomes, as indicated by the absence of LIMP-II or LC3 (Fig. 6a).

## Discussion

The ability of *Salmonella* to successfully establish an infectious niche and replicate in a wide variety of cell types is linked to the pathogen’s capability of residing in a vacuolar compartment and evasion of phago-lysosomal clearance [28, 29]. Our current knowledge of the SCV biogenesis, maturation and interactions with host proteins is largely based on immunofluorescence/co-localization studies, which is limited by the choice of antibodies [21]. A number of prior studies using gradient centrifugation, immunoprecipitation, or FACS sorting have provided important insights into a number of host proteins with which *Salmonella* effector proteins interact at the SCV interface [12–14], but a complete characterization has remained elusive due to technical difficulties in the purification. Here we report a rapid, new method to isolate intact SCVs by labeling *Salmonella* with para-magnetic nanoparticles prior to infection. After gentle disruption of the cell membrane, the SCV can be easily purified using a magnetic field.

The method described here for SCV isolation using paramagnetic nanoparticles is rapid and requires a minimum of manipulations, in contrast to column- or flow-assisted cell sorting (FACS)-based methods [14]. This method presents several advantages when compared to the traditional phagosome isolation methods. (i) The process eliminates time-consuming, multiple centrifugation steps, allowing more samples to be handled in less time and yields intact SCVs at high purity. (ii) The method also eliminates the need for specialized and sensitive equipment, such as ultracentrifugation or flow-assisted cell sorting. (iii) The approach also avoids the necessity of covalent labeling of the bacterial outer membrane with ligands using chemical reagents, which may affect the secretion of virulence proteins necessary for establishment of the intracellular niche. As demonstrated in Fig. 3a, the labeling of the nanoparticles does not interfere with *Salmonella*’s ability to invade either intestinal epithelial or macrophage cells. The SCVs purified using paramagnetic nanoparticles showed association with standard SCV markers, Rab5 and LAMP-1 [20, 21, 25], but essentially no association with another lysosomal marker, LIMP-2, confirming minimal or no contamination with lysosomes or other cellular compartments. Furthermore, we could demonstrate the presence of *Salmonella* effector proteins known to co-localize with SCVs. We suggest the method described here could also be applied for isolation of phagosomes harboring other intracellular bacteria such as *Mycobacteria*, *Legionella*, *Brucella*, etc. This should aid in identification and characterization of host factors associated with the membranes of such intracellular pathogens and which could potentially serve as pharmaceutical targets against intracellular pathogens residing within vacuoles.

## Conclusion

In summary this, method for the isolation of *Salmonella*-containing vacuoles (SCV) is specific, rapid, efficient, requires a minimum of specialized equipment, and can be easily adapted to address key biological questions concerning host–pathogen interactions.

## Methods

### Cell culture and growth conditions

Human macrophage like cells THP-1 (DSMZ Cat. Nr. ACC-16 (Tsuchiya et al. [30]) were grown in Iscove’s Dulbecco modified Eagle medium (Biochrom), supplemented with 10% fetal Calf serum (FCS) under standard tissue culture conditions of 37 °C, 5% CO_2_. The cells were regularly passaged with 1% trypsin EDTA (Biochrom).

The wild-type, virulent *S. typhimurium* strain SL1344 (Hoiseth and Stocker [31]) harboring the GFP fusion under control of the *rpsM* promoter [4] was streaked onto LB agar plates containing kanamycin (50 μg/ml) the day before the assays, and a single colony was used to inoculate in LB broth culture was grown to the late log phase (OD_600_ ~ 2). The cells were centrifuged and the resulting pellets were re-suspended in cell culture medium and diluted to obtain the desired MOI.

### SCV isolation

#### Labeling *Salmonella* with paramagnetic nanoparticles

From a stock solution of 3 mg/ml of carboxyl-coated, paramagnetic cobalt nanoparticles of diameter 10–50 nm (TurboBeads, Zurich), a 0.5 ml aliquot was sonicated to disperse aggregates, followed by centrifugation at 1000×*g* for 30 s. Following centrifugation, 0.1 ml of the resulting supernatant was diluted 1:10 in sterile, de-ionized water prior to use. *Salmonella* strains grown in LB as described above were collected from 1 ml of culture by centrifugation in a microfuge at 16,000×*g* for 5 min. The resulting bacterial pellets were re-suspended in PBS and incubated in the presence of a 5:1 ratio of nanoparticles:bacteria at 37 °C for 20 min, with shaking. The bacterial suspension was then filtered through a 0.2 μm filter fitted to a 5 ml syringe. The filters were washed twice with two volumes of PBS, then the filter was inverted and the bacteria recovered in 1 ml of PBS collected into a sterile microfuge tube, and used either for infection or processed for electron microscopy.

#### Infection and recovery of bacteria

THP-1 human macrophage cells grown in a 6-well cell culture plate were infected at a cell density of 10^6^ cells/well with the nanoparticle-labeled *Salmonella* (MOI = 5) and incubated at 37 °C, 5% CO_2_. 24 h post-infection, the cell culture media was removed; infected cells were washed twice with PBS then lysed by addition of 1 ml/well of cell lysis buffer (0.1% Triton X-100 in water). The resulting cell lysates were then placed in a magnetic field (Single Place Magnetic Stand, Ambion, USA) and the supernatant was carefully removed and discarded, and replaced by 1 ml of a 1× PBS/2% sucrose solution. The buffer was again removed and replaced by 0.1 ml of the same buffer to concentrate the SCVs, which were then used either for staining with antibodies or subjected to SDS PAGE and Western blotting.

#### Western blots

Isolated SCV’s were centrifuged and pelleted, followed by boiling the samples in Laemmli (6×) buffer. Equal volumes were loaded onto 12% SDS PAGE and transferred to nitrocellulose membrane (GE Healthcare). The membrane was blocked in 3% skim milk powder in 1× TBS, followed by incubation in appropriate primary antibodies and HRP conjugated secondary antibodies at the above mentioned dilutions. Signals were revealed using the ECL detection system kit (Thermo scientific pierce).

#### Confocal microscopy

THP-1 cells were seeded onto 12 mm sterile coverslips (Carl Roth), infected with *Salmonella* strains expressing GFP at an MOI of 5. 24 h post-infection, the cells were washed in pre-warmed 1× PBS and fixed with PBS/4% paraformaldehyde (PFA, Sigma) for 15 min, permeabilized with 0.1% Triton X-100 (Sigma-Aldrich) in PBS for 5 min, blocked with PBS/1.5% BSA (sigma) for 1 h and incubated with the appropriate antibodies for 1 h or overnight at 4 °C. The cells were washed with 1× PBS and incubated with appropriate secondary antibodies for 1 h at room temperature. The coverslips were mounted with Mowiol (Sigma-Aldrich) onto glass slides (Carl Roth) and were visualized using a Leica SP-II laser scanning confocal microscope (LCSM) using a 63× oil immersion objective.

Isolated SCVs were incubated for 1 h with specific primary antibodies in 1.5 ml microfuge tubes (Eppendorf) and placed in a magnetic stand. The supernatant was removed by pipetting along the opposite wall of the of the microfuge tube (relative to the magnetic stand), and the SCVs were gently washed with 1× PBS/2% sucrose solution, followed by incubation with suitable secondary antibody in the same microfuge tubes for 1 h. After re-placement in the magnetic stand, the samples were again washed and mounted onto glass slides for microscopic visualization.

#### Transmission electron microscopy

THP-1 cells were infected with either tagged or non-tagged *Salmonella*, and the cells were then immediately fixed by addition of 1.5% PFA and 1.5% glutaraldehyde in 0.15 M sodium cacodylate buffer, pH 7.4. After incubation at room temperature for 1 h, the cell suspensions were post-fixed for 2 h at 4 °C in 1% osmium tetroxide in sodium cacodylate buffer, and subsequently dehydrated in a series of ethanol steps, and then further processed with acetone prior to Epon embedding. Sections were cut with a microtome and mounted on Formvar-coated copper grids. The sections were post-fixed with uranyl acetate and lead citrate and examined under the electron microscope.

## Additional file

**Additional file 1: Figure S1.** In vitro grown, nanoparticle-labeled *Salmonella* does not show exterior labeling with host cell lysosomal marker antibodies. Paramagnetic nanoparticle-tagged, GFP-labeled *Salmonella* were incubated with anti-Rab5, LAMP-1 and LIMP-II antibodies for 1 h at room temperature, followed by a wash with 1× PBS, and subsequently stained with the Alexa-Flour 594 labelled secondary antibodies. In vitro, culture-grown GFP-expressing *Salmonella* does not show labelling with any of the host cell markers.

## Abbreviations

SCV: Salmonella-containing vacuole
MOI: multiplicity of Infection
CFU: colony forming unit
LAMP1: lysosomal associated membrane protein 1
LC3: microtubule-associated protein 1A/1B-light chain 3
