# Supplementary figures and images for: Rapid Isolation of intact Salmonella-containing vacuoles using paramagnetic nanoparticles
Source: Gut Pathog. 2018 Jul 31;10:33. doi: 10.1186/s13099-018-0256-7 (PMC6069567; doi:10.1186/s13099-018-0256-7)

Additional file 1.

Figure S 1.

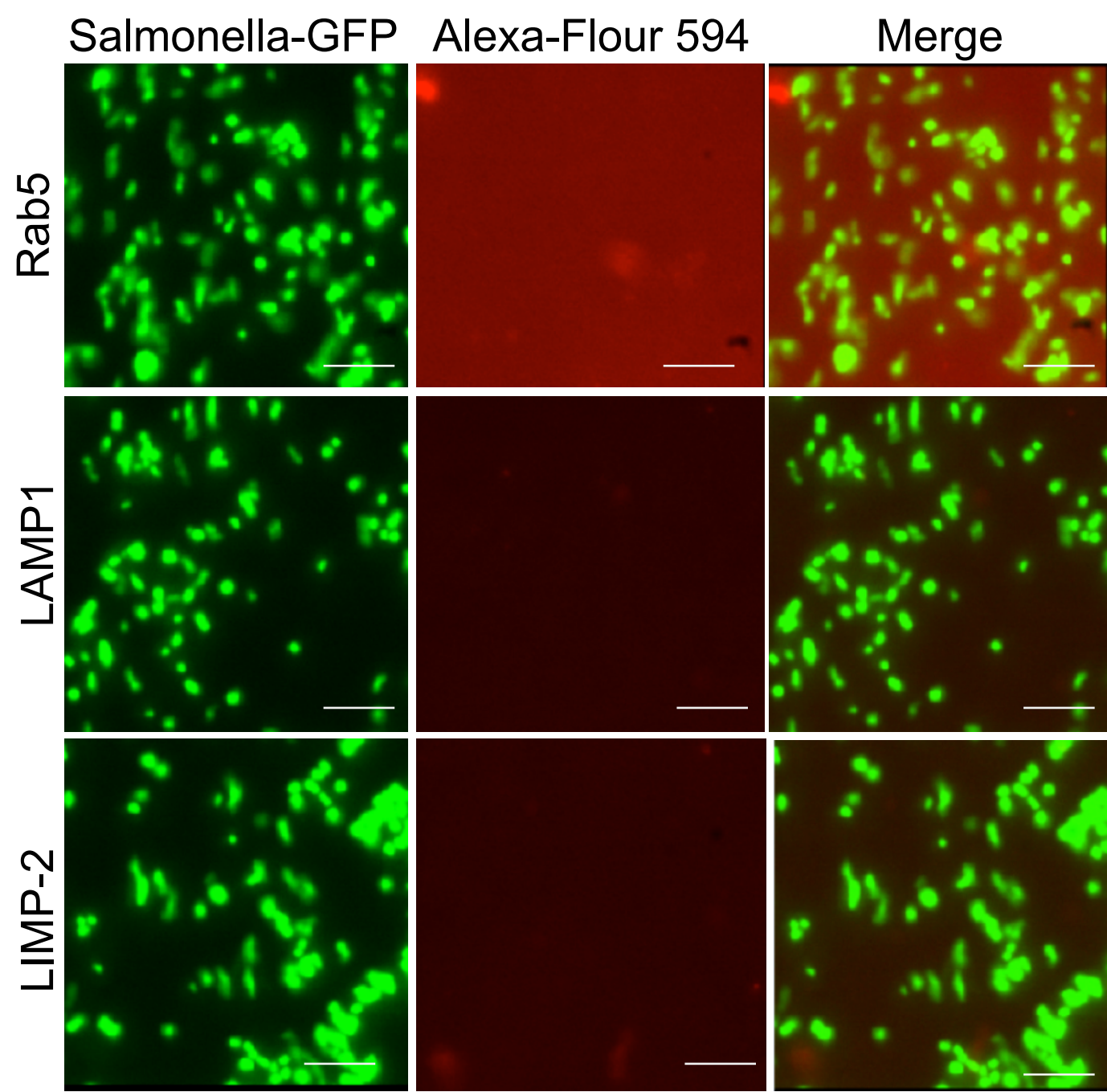

Supplement: Supplementary file 1 — Additional file 1: Figure S1. In vitro grown, nanoparticle-labeled Salmonella does not show exterior labeling with host cell lysosomal marker antibodies. Paramagnetic nanoparticle-tagged, GFP-labeled Salmonella were incubated with anti-Rab5, LAMP-1 and LIMP-II antibodies for 1 h at room temperature, followed by a wash with 1× PBS, and subsequently stained with the Alexa-Flour 594 labelled secondary antibodies. In vitro, culture-grown GFP-expressing Salmonella does not show labelling with any of the host cell markers. [file 13099_2018_256_MOESM1_ESM.pdf]
